# Validation of self-reported diabetes in a representative sample of São Paulo city

**DOI:** 10.1590/S1518-8787.2017051006378

**Published:** 2017-03-07

**Authors:** Mariane de Mello Fontanelli, Juliana Araújo Teixeira, Cristiane Hermes Sales, Michelle Alessandra de Castro, Chester Luiz Galvão Cesar, Maria Cecilia Goi Porto Alves, Moisés Goldbaum, Dirce Maria Marchioni, Regina Mara Fisberg

**Affiliations:** I Programa de Pós-Graduação de Nutrição em Saúde Pública. Universidade de São Paulo. São Paulo, SP, Brasil; II Departamento do Epidemiologia. Faculdade de Saúde Pública. Universidade de São Paulo. São Paulo, SP, Brasil; III Instituto de Saúde. Secretaria de Estado da Saúde. São Paulo, SP, Brasil; IV Departamento de Medicina Preventiva. Faculdade de Medicina. Universidade de São Paulo. São Paulo, SP, Brasil; V Departamento de Nutrição. Faculdade de Saúde Pública. Universidade de São Paulo. São Paulo, SP, Brasil

**Keywords:** Diabetes Mellitus, epidemiology, Diagnostic Self Evaluation, Health Surveys, Reproducibility of Results, Validation Studies

## Abstract

**OBJECTIVE:**

To validate the self-reported diabetes mellitus in adults and older adults living in the city of São Paulo, Brazil.

**METHODS:**

We have used data of 569 subjects (284 adults and 285 older adults), participants of the population-based cross-sectional study *Inquérito de Saúde do Município de São Paulo* (Health Survey of São Paulo). Fasting glucose ≥ 7.0 mmol/L (126 mg/dL) and/or use of drugs (oral hypoglycemic and/or insulin) defined the diagnosis of diabetes mellitus. We have validated the self-reported diabetes mellitus by calculating the sensitivity, specificity, positive predictive values, and negative predictive values. We have used Poisson regression with robust variance to verify the factors associated with the sensitivity of the self-reported datum. For all analyses, we have considered the sample design of the study.

**RESULTS:**

The sensitivity of self-reported diabetes mellitus was 63.8% (95%CI 49.2–76.3), specificity was 99.7% (95%CI 99.1–99.9), positive predictive value was 95.5% (95%CI 84.4–98.8), and negative predictive value was 96.9% (95%CI 94.9–98.2). The correct reporting of diabetes mellitus was more prevalent among older adults (PR = 2.0; 95%CI 1.2–3.5) than among adults.

**CONCLUSIONS:**

The use of the datum of self-reported diabetes mellitus is valid, especially among older adults living in the city of São Paulo. The results highlight the need to track diabetes mellitus in asymptomatic subjects who have one or more risk factors for it, mainly in the adult population of this city.

## INTRODUCTION

Diabetes mellitus (DM) is a heterogeneous group of metabolic disorders characterized by hyperglycemia caused by defects in insulin action or insulin secretion^1^. More than 90.0% of the cases of DM correspond to type 2 DM, whose prevalence is increasing, especially in developing countries^3^. Data from 133 studies of 91 countries suggest that the overall prevalence of DM, estimated as 6.4% in 2010, will be 7.7% in 2030, reaching 439 million persons and representing a significant impact on national health systems^18^.

Data from 27 Brazilian cities assessed in the *Vigilância de fatores de risco e proteção para doenças crônicas por Inquérito Telefônico* (VIGITEL – Risk and Protective Factors Surveillance for Chronic Diseases by Telephone Interviews) showed a prevalence of 6.9% (95%CI 6.5–7.3) for self-reported DM^a^, with a growth of 0.21 percentage points per year for the 2006-2013 period when prevalence rates were monitored. Data from the same study suggest the prevalence of 8.2% (95%CI 6.9–9.6) for self-reported DM in the city of São Paulo in 2013^a^. Even more alarming figures were presented by the *Estudo Longitudinal de Saúde do Adulto* (ELSA-Brazil – Brazilian Longitudinal Study of Adult Health), conducted in six Brazilian cities with 15,102 civil servants aged 35-74 years, whose prevalence of DM was 19.7% (95%CI 19.0–20.3), determined by fasting glucose test, oral glucose tolerance test, and glycated hemoglobin test^19^.

In this scenario, the use of information on self-reported DM becomes an important and convenient tool for population monitoring and surveillance, because of its low-cost and speed in data collection and analysis^9^.

Self-reported DM has often been used in national and international surveys^14,16,19^. Some studies have been conducted in order to validate this self-reported information using biochemical tests, for specific locations and populations^5,9,13,17,20^. However, there is no consensus on the precision and accuracy of this datum^20^. Thus, the purpose of this study was to validate self-reported DM in adults and older adults living in the city of São Paulo, Brazil.

## METHODS

We have used data from the *Inquérito de Saúde de São Paulo* (ISA – Health Survey of São Paulo), a cross-sectional population-based study with probabilistic sampling of the residents of the urban area of this city.

Briefly, the sample was calculated by conglomerates in two stages: census tract (primary sampling units) and household (secondary sampling units). In the first stage, 70 census sectors were randomly selected from the register of the *Pesquisa Nacional por Amostra de Domicílios* (PNAD – National Household Survey) of 2005 of the Brazilian Institute of Geography and Statistics (IBGE). Domains of study were set according to age and sex. In order to preserve the representativeness of each domain, different sampling fractions were applied, considering the participation of age groups in the population of the urban area of São Paulo. Details of sampling are described in Alves and Escuder^b^.

The study involved 2,086 adults (20-59 years) and older adults (60 years or older) of both sexes. Of these, 1,662 had two 24-hour dietary recalls (24hR) collected for the analysis of food intake and 592 had blood samples taken for the analysis of biochemical data. There was no difference in terms of sexes, age, income, and education between the original ISA sample and the remaining sample^18^.

For this study, 569 individuals were selected (284 adults and 285 older adults), aged 20 years or older, who had their fasting glucose evaluated or who reported the use of oral hypoglycemic agents or insulin. Twenty-three individuals were excluded because they had no values for the analysis of glucose.

In 2008, information was obtained in-home, using a structured questionnaire administered by trained interviewers, who collected demographic, socioeconomic, lifestyle, health conditions, and health care service data.

The first 24hR was collected in the first home visit and conducted using the Multiple-Pass method^c^, in which the respondent is guided through five steps (quick list, forgotten foods, time & occasion, detail cycle, and final probe) in a standardized process, which helps to maintain the individuals interested and engaged in the interview, and helps them remember all the items consumed. The second 24hR was conducted by telephone, two or three days before the second home visit, using the interview system of the Nutrition Data System for Research (NDS-R), version 2007, developed by the Nutrition Coordinating Center at the University of Minnesota, Minneapolis, MN, USA, which resembles the computerized version of the Multiple-Pass method (the Automated Multiple-Pass Method) as it enables the same structure to collect dietary data in five steps.

In the second home visit, we measured the anthropometric and blood pressure parameters, collected venous blood, and investigated drug use. The data were collected by a previously trained nursing technician, according to the recommendations for measuring weight, height^20^, waist circumference^7^, and blood pressure^17^.

For blood collection, participants were instructed to fast for 12 hours, without eating or drinking (for alcoholic beverages, the minimum fast should be 36 hours), and no physical activity or exhaustive physical efforts should be performed during the 24 hours preceding the test. Blood samples were collected in vacuum tubes with a clot activator (BD Vacutainer, # 368660, Franklin Lakes, NJ, USA), packed in coolers, and immediately transported to the laboratory for processing.

The self-reported DM information was obtained from the structured questionnaire from the answer to the question: “Do you have a chronic disease, a long-term disease, or a recurring disease?” Individuals who answered “Diabetes (if only gestational diabetes, select no)” were considered as self-reporting DM. A subsequent question was made: “Who told you that you have diabetes?” All individuals answered that a doctor made the diagnosis.

The laboratory diagnosis was made by measuring fasting serum glucose, using the enzyme glucose oxidase method (Glucose Liquiform, Labtest, Lagoa Santa, Minas Gerais, Brazil) and an automated system (LabMax 240, Lagoa Santa, Minas Gerais, Brazil). All biochemical analyses were carried out in duplicate with a 10% variation limit set as the criterion for repetition of the test.

The use of drugs was investigated by asking the following question on the day of blood collection: “Do you currently use any medication and/or supplement?” “Which one?” The use of medication was also considered when the individual selected the options “routinely use insulin” or “routinely take oral medication” for the question “What do you do to control your diabetes?” (question included in the structured questionnaire). The individual was classified as having DM if the value of fasting glucose was ≥ 7 mmol/L (126 mg/dL) or if he or she used medications (oral hypoglycemic agents or insulin), according to current criteria at the time of the study^d^.

Prediabetes was characterized by fasting glucose levels between 5.6 mmol/L (100 mg/dL) and 6.9 mmol/L (125 mg/dL) and if no oral hypoglycemic agents or insulin were used^d^.

Alcoholism was investigated by a specific questionnaire to assess alcohol dependence, named CAGE (Cut down, Annoyed by criticism, Guilty and Eye-opener)^6^. The cutoff point adopted for positivity of the test was two or more positive answers. Usual alcohol consumption was estimated by incorporating 24hR data in the Multiple Source Method program.

Physical activity data was collected using the International Physical Activity Questionnaire (IPAQ), long version^4^. For the analyses, physical activity of leisure was considered, classified either as sufficient (practice of physical activity for at least 30 minutes a day, five days a week, of moderate intensity, or at least 20 minutes a day, three days a week, of vigorous intensity) or insufficient.

Body mass index (BMI) was calculated as weight divided by the square of height (BMI = weight [kg] / height [m]^2^) and classified according to the World Health Organization^20^ for adults and the Pan American Health Organization (PAHO)^10^ for older adults. The BMI data were categorized into overweight (overweight and obesity) and not overweight (underweight and normal weight).

Waist circumference was classified as adequate or inadequate according to the cutoff points of 88 cm for women and 102 cm for men^7^.

Hypertension was defined as systolic blood pressure ≥ 140 mmHg and diastolic blood pressure ≥ 90 mmHg or the use of drugs for hypertension^17^.

The study sample design was considered for all statistical analyses by using survey module commands available on Stata version 12.0. The significance level was set at 5%.

The prevalence of DM was calculated for the total population and by age group (adults and older adults). Differences between the relative frequencies of socioeconomic and lifestyle variables according to the presence of DM were analyzed using Pearson’s Chi-square test. Absolute and relative frequencies were used to compare diagnosed DM and self-reported DM.

The validation of self-reported DM was determined by sensitivity (proportion of individuals with DM that self-reported the condition), specificity (proportion of individuals without DM who reported not having the condition), positive predictive value (proportion of individuals who self-reported DM and were classified as having the condition), and negative predictive value (proportion of individuals who self-reported not having DM and were classified as such). The diagnosis of DM (fasting glucose ≥ 7 mmol/L (126 mg/dL) or the use of oral hypoglycemic agents or insulin) was considered as reference for these calculations. The validation of the self-reported data was also determined according to the socioeconomic and lifestyle variables.

The univariate Poisson regression with robust variance was used to identify factors associated with the sensitivity of self-reported DM, so that the model could determine the probability of correct answer given the presence of DM.

The University Research Ethics Committee (CAAE 26800414.1.0000.5421) and the Ethics Committee of the Municipal Health Secretariat of São Paulo (CAAE 003.0.162.000-08) have approved this study. The objectives of the study were explained to all potential participants and we emphasized that their participation would be completely voluntary. All those who agreed to participate in the study, before starting any procedure, signed the written informed consent.

## RESULTS

The prevalence of DM among residents in the urban area of the city of São Paulo was estimated at 8.0% (95%CI 6.1–10.6), 5.0% in adults (95%CI 3.0–8.2) and 20.7% in older adults (95%CI 16.1–26.1). The prevalence of prediabetes in the study population was 4.4% (95%CI 2.7–7.3).

Of the individuals with DM, 79.5% (95%CI 64.0–89.4) were using oral hypoglycemic agents (71.9% of adults and 87.0% of older adults) and 11.1% (95%CI 4.7–24.1) were using insulin (11.9% of adults and 10.3% of older adults). Of the individuals who self-reported DM, the older adults accounted for most of those who reported routine medical visits for DM care – 61.3% (95%CI 40.4–78.7) for older adults against 38.7% (95%CI 21.3–59.6) for adults.

The sample studied comprised subjects that were predominantly female (53.4%), adults (80.7%), who self-reported as white (51.6%), with head of household with six or more years of education (72.7%), with family income *per capita* of more than one minimum wage (62.4%), without partner (52.6%), and who possibly do not abuse alcohol (89.2%). Among the individuals with DM, there was a predominance of older adults, with head of household with up to five years of education, former smokers, overweight, with inadequate waist circumference, and hypertensive (Table 1).

**Table 1:** Baseline characteristics of adults and older adults living in São Paulo, SP, Brazil, according to the diagnosis of diabetes mellitus, 2008.

Characteristics	With DM		Without DM		p^b^
	
	n	%	n	%	
Sex (n = 569)					
Male	28	6.4	188	93.6	0.128
Female	54	9.5	299	90.5	
Age group (n = 569)					
Adults	17	5.0	267	95.0	0.000
Older adults	65	20.7	220	79.3	
Self-reported race (n = 569)					
White	49	7.6	306	92.4	0.623
Non-white	33	8.8	181	91.2	
Education of head of household (n = 566)					
Up to 5 years	57	15.0	196	85.0	0.001
6 or more years	25	5.5	288	94.5	
Family income *per capita* (n = 569)					
Up to 1 MW^a^	31	7.3	208	92.7	0.620
More than 1 MW	51	8.5	279	91.5	
Marital status (n = 269)					
With partner	5	3.3	109	96.7	0.097
Without partner	19	8.4	136	91.6	
Smoking status (n = 569)					
Non-smoker	44	6.2	276	93.8	0.013
Former smoker	29	15.4	118	84.6	
Current smoker	9	6.8	93	93.2	
Alcohol abuse (n = 255)					
Possibly no	21	3.9	212	96.1	0.111
Possibly yes	4	9.2	18	90.8	
Physical activity of leisure (n = 569)					
Sufficient	4	4.5	50	95.5	0.229
Insufficient	78	8.5	437	91.5	
BMI (n = 545)					
Not overweight	33	4.8	239	95.2	0.028
Overweight	44	10.7	229	89.3	
Waist circumference (n = 489)					
Adequate	18	3.9	192	96.1	0.001
Inadequate	56	13.54	223	86.5	
Hypertension (n = 569)					
No	22	3.7	283	96.3	0.000
Yes	60	17.4	204	82.6	
Health insurance (n = 569)					
No	49	7.1	321	92.9	0.342
Yes	33	9.6	166	90.4	
Hospitalization in the past year (n = 569)					
No	77	8.3	448	91.7	0.160
Yes	5	4.3	39	95.7	
Health problem in the past 15 days (n = 569)					
No	59	7.5	378	92.5	0.303
Yes	23	10.3	109	89.7	

DM: Diabetes mellitus; BMI: body mass index

^a^ MW: minimum wage (US$260.00).

^b^ Pearson’s Chi-square test. Survey module commands were considered for analyses.

Data from this study shows that 2.9% (95%CI 1.7–4.9) of the population of adults and older adults of São Paulo in 2008 were unaware of having DM. Of the individuals with DM, 63.8% self-reported the condition, while 36.2% were unaware of having the disease (Table 2). Adults corresponded to 80.4% (95%CI 57.9–92.5) of these individuals.

**Table 2:** Self-reported and diagnosed diabetes mellitus in adults and older adults living in São Paulo, SP, Brazil, 2008.

Self-reported diabetes mellitus	Diagnosed diabetes mellitus						Total		

	Yes			No					
		
	n	%	95%CI	n	%	95%CI	N	%	95%CI
Yes	63	63.8	49.2–76.3	4	0.3	0.0–0.9	67	5.4	4.0–7.2
No	19	36.2	23.7–50.8	483	99.7	99.1–99.9	502	94.6	92.8–96.0
Total	82	100	-	487	100	-	569	100	-

* Diagnosis by fasting blood glucose (≥ 7 mmol/L [126 mg/dL]) or use of oral hypoglycemic agents or insulin. Survey module commands were considered for analyses.

Table 3 shows the validation of self-reported DM. When the population was stratified by socioeconomic and lifestyle characteristics, the sensitivity was higher among older adults when compared to adults, and the negative predictive value was higher among the non-hypertensive when compared to the hypertensive (Table 4). For other categories, there was no difference regarding specificity and positive predictive values.

**Table 3:** Sensitivity, specificity, positive predictive values, and negative predictive values of self-reported diabetes mellitus*. São Paulo, SP, Brazil, 2008.

Validity	%	95%CI
Sensitivity	63.8	49.2–76.3
Specificity	99.7	99.1–99.9
Positive predictive value	95.5	84.4–98.8
Negative predictive value	96.9	94.9–98.2

* Survey module commands were considered for analyses.

**Table 4:** Validation of self-reported diabetes mellitus according to baseline characteristics. São Paulo, SP, Brazil, 2008.

Characteristics	SENS (%)	95%CI	SPEC (%)	95%CI	PPV (%)	95%CI	NPV (%)	95%CI
Sex (n = 569)								
Male	72.2	47.2–88.3	99.7	97.8–100.0	94.0	65.7–99.2	98.1	95.5–99.2
Female	58.9	40.8–74.8	99.8	99.1–100.0	96.6	86.3–99.3	95.9	92.8–97.7
Age group (n = 569)								
Adults	42.1	22.4–64.6^b^	97.9	95.7–99.0	100.0	-	97.0	94.4–98.4
Older adults	85.8	70.7–93.8^b^	98.4	94.6–99.6	93.4	78.4–98.3	96.4	91.8–98.4
Self-reported race (n = 569)								
White	74.3	53.2–88.1	99.7	99.0–99.9	94.8	83.3–98.5	97.9	95.6–99.1
Non-white	49.3	31.3–67.5	99.9	98.9–100.0	97.0	79.8–99.6	95.3	90.4–97.8
Education of head of household (n = 566)								
Up to 5 years	71.7	51.2–86.0	99.3	98.1–99.8	95.0	84.3–98.5	95.2	88.3–98.1
6 or more years	55.7	34.3–75.2	99.9	99.1–100.0	96.3	75.5–99.5	97.5	95.3–98.7
Family income *per capita* (n = 569)								
Up to 1 MW^a^	79.0	57.9–91.2	99.3	97.8–99.8	90.0	68.3–97.4	98.4	96.0–99.4
More than 1 MW	56.0	39.4–71.3	100	-	100	-	96.1	92.9–979
Marital status (n = 269)								
With partner	40.6	9.6–81.5	99.6	97.5–100.0	79.5	26.9–97.6	98.0	91.5–99.6
Without partner	49.3	22.7–76.3	99.8	98.5–100.0	95.8	71.7–99.5	95.5	90.1–98.1
Smoking status (n = 569)								
Non-smoker	58.1	37.5–76.2	99.6	98.5–99.9	89.7	68.2–97.2	97.3	94.7–98.7
Former smoker	77.6	55.1–90.7	100	-	100	-	96.1	90.7–98.4
Current smoker	51.8	17.5–84.5	100	-	100	-	96.6	89.5–99.0
Alcohol consumption (n = 566)								
1st tertile	74.8	49.0–90.2	99.5	97.9–99.9	95.6	82.4–99.0	96.4	90.7–98.7
2nd tertile	43.4	18.7–71.9	99.7	98.2–100.0	93.9	62.7–99.3	94.9	89.2–97.7
3rd tertile	85.8	53.2–97.0	99.9	99.1–100.0	96.8	78.3–99.6	99.3	96.8–99.9
Physical activity of leisure (n = 569)								
Sufficient	19.2	3.3–62.6	100	–	100	–	96.4	86.1–99.1
Insufficient	67.0	52.0–79.2	99.7	99.0–99.9	95.4	84.1–98.8	97.0	95.1–98.2
BMI (n = 545)								
Not overweight	72.1	48.3–87.8	99.6	98.1–99.9	91.0	65.6–98.2	98.4	95.7–99.4
Overweight	59.9	40.0–76.9	99.9	99.2–100.0	98.3	87.9–99.8	95.7	91.4–97.9
Waist circumference (n = 489)								
Adequate	67.3	37.1–87.8	99.7	97.9–100.0	90.0	51.6-98.7	98.7	95.9–99.6
Inadequate	60.8	42.5–76.4	99.9	98.9–100.0	98.5	89.5-99.8	94.2	89.3–96.9
Hypertension (n = 569)								
No	75.0	50.5–89.8	100	-	100	-	99.1	97.5–99.6*
Yes	58.7	41.9–73.7	99.1	96.9–99.7	93.1	77.1–98.2	91.9	85.4–95.7*
Health insurance (n = 569)								
No	59.9	42.5–75.1	99.7	98.6–99.9	93.4	73.3–98.7	97.0	94.2–98.5
Yes	68.9	46.0–85.2	99.9	98.8–100.0	97.9	85.1–99.8	96.8	93.2–98.5
Hospitalization in the past year (n = 569)								
No	62.5	47.3–75.5	99.8	99.3–100.0	97.2	88.2–99.4	96.7	94.5–98.1
Yes	100	-	98.2	92.5–99.6	74.2	34.5–94.0	100	-
Health problem in the past 15 days (n = 569)								
No	61.5	44.6–76.0	99.9	99.2–100.0	97.8	84.7–99.7	97.0	94.6–98.3
Yes	71.5	37.8–91.2	99.1	97.0–99.7	89.7	68.8–97.2	96.8	89.8–99.1

SENS: sensitivity, SPEC: specificity, PPV: positive predictive value, NPV: negative predictive value, BMI: body mass index

^a^ MW: minimum wage (US$260.00).

^b^ Significant difference according to 95%CI. Survey module commands were considered for analyses.

In the univariate Poisson regression model, only the age group was associated with sensitivity (Table 5). The prevalence of the correct answer for DM was higher among older adults (PR = 2.0; 95%CI 1.2–3.5) when compared to adults. The absolute number of individuals with DM (n = 82) did not allow us to perform multiple Poisson regression analysis.

**Table 5:** Prevalence ratios of the sensitivity of self-reported diabetes mellitus according to baseline characteristics (raw data). São Paulo, SP, Brazil, 2008.

Characteristics	Univariate model	

	PR	95%CI
Sex (n = 82)		
Male	1.00	-
Female	0.81	0.53–1.25
Age group (n = 82)		
Adults	1.00	-
Older adults	2.04	1.18–3.52
Education of head of household (n = 82)		
Up to 5 years	1.00	-
6 or more years	0.78	0.49–1.23
Family income *per capita* (n = 82)		
Up to 1 MW*	1.00	-
More than 1 MW	0.71	0.50–1.01
Grams of alcohol consumption (tertiles) (n = 81)		
1st tertile	1.00	-
2nd tertile	0.58	0.27–1.24
3rd tertile	1.15	0.78–1.68
Physical activity of leisure (n = 82)		
Sufficient	1.00	-
Insufficient	0.29	0.06–1.43
BMI (n = 77)		
Not overweight	1.00	-
Overweight	0.83	0.53–1.30
Waist circumference (n = 74)		
Adequate	1.00	-
Inadequate	0.90	0.54–1.52
Hypertension (n = 82)		
No	1.00	-
Yes	0.78	0.53–1.14

* MW: minimum wage (US$260.00). Survey module commands were considered for analyses.

## DISCUSSION

The results indicate that, in order to use the self-reported DM datum for residents of the city of São Paulo, we need to consider the age group of the study population, as, while the sensitivity of this information is 85.8% (95%CI 70.7–93.8) in older adults, the same is only 42.1% (95%CI 22.4–64.6) in adults, even though the sensitivity in the study population is 63.8% (95%CI 49.2–76.3).

A Brazilian study that has assessed the self-reported DM information in older adults of the city of Bambuí, state of Minas Gerais, Brazil, had a sensitivity rate of 57.1% (95%CI 50.3–63.8)^11^. In international studies that have investigated self-reported DM among adolescents, adults and older adults, the sensitivity ranged from 30.1% (95%CI 24.0–36.2) to 70.4% (95%CI 64.5–75.8)^5,9,13,20^. The results show that, as in this study, sensitivity depends on the study population.

One of the causes of the variation of sensitivity is the criterion that defines DM for validation of self-reported data. Schneider et al.^20^ has found that the definition of DM as fasting glucose ≥ 7.8 mmol/L (140 mg/dL) or the use of drugs had higher sensitivity than the definition of fasting glucose ≥ 7.0 mmol/L (126 mg/dL) or the use of drugs in participants of the Atherosclerosis Risk in Communities Study. Despite this finding, in our study, the classic definition of fasting glucose ≥ 7.0 mmol/L (≥ 126 mg/dL) was chosen to be used as diagnosis, which has been indicated since 1997 by the Expert Committee on the Diagnosis and Classification of Diabetes Mellitus and accepted by the World Health Organization and the Brazilian Diabetes Society^23^.

The difference found in the sensitivity of self-reported DM among adults and older adults in this study may have occurred because, in the population studied, older persons tend to the visit medical care more often than adults and, therefore, had already been diagnosed with DM. Another possible reason is that older adults have more severe cases of the disease, since age is an important risk factor for the development of this condition^12,15^. On the other hand, of the individuals classified with DM and who did not know about the condition, most were adults, a fact that may be related to the initial stage of the disease, when it has not yet been diagnosed.

Prediabetes and risk factors related to this condition were present in a substantial part of the study population. This fact emphasizes the importance of screening to minimize the progression of DM, condition that accounted for 5.4% of Brazilian disability-adjusted life years (DALY) in 2008^8^.

The mass screening of individuals who do not show symptoms of DM or prediabetes is not recommended; however, the American Diabetes Association suggests tracking the diagnosis of DM by fasting glucose, glycated hemoglobin, or by glucose tolerance test of individuals that are overweight (BMI ≥ 25 kg/m^2^) and who have one of the following risk factors: physical inactivity, first-degree relative with DM, high-risk race/ethnic group (African American, Latino, Native American, Asian American, and Pacific Islanders), women who have been diagnosed with gestational DM, hypertension, HDL-c < 0.9 mmol/L (35 mg/dL) or level of triglycerides > 2.8 mmol/L (250 mg/dL), women with polycystic ovary syndrome, glycated hemoglobin ≥ 5.7%, impaired glucose tolerance or impaired fasting glucose on previous testing, history of cardiovascular disease, or other conditions associated with insulin resistance. Among those who do not present the mentioned risk factors, the screening of DM should be started in individuals aged 45 years and repeated every three to five years^1^.

The screening of DM in Brazilian primary care is recommended when asymptomatic individuals have sustained blood pressure greater than 135/80 mmHg^e^. In this study, 57.9% of the individuals who were unaware of having the disease met this criterion (data not shown).

Data from this study shows that 2.9% of the adults and older adults of São Paulo in 2008 were unaware of having DM. Similar result was found in a multicenter study conducted in 1992, which described the prevalence of 3.4% of undiagnosed DM in a population aged 30-69 years in Brazilian urban areas^12^. However, when individuals with DM are taken as total, the proportion of individuals unaware of the condition is 36.2%. Data from ELSA-Brazil shows an even higher prevalence (50.4%) of undiagnosed cases of DM^15^, a difference that may have occurred because of the age of the population studied, 35-74 years, while this study, which uses data from the ISA, included individuals aged 20-94 years.

Age group was associated with sensitivity of self-reported DM in the univariate analysis. Among older adults living in Minas Gerais, Brazil, Lima-Costa et al. have detected that visiting a doctor within the previous two years and educational level presented a positive association with sensitivity of self-reported DM^11^. Educational level has been described as a marker of social differences in health. Data from the PNAD shows a higher prevalence of investigated chronic diseases, including DM, in segments of the population with low literacy levels^2^, which corroborates the higher prevalence of persons with head of household with up to five years of education among the individuals with DM found in this study.

The specificity of self-reported DM in a national study was 96.0% (95%CI 94.7–97.0). In international studies, the specificity of this information ranged from 96.8% (95%CI 96.4-97.2) to 99.4% (95%CI 99.2–99.7)^5,9,13,16^; therefore, the result of this study is consistent with the literature (99.7%; 95%CI 99.1–99.9). Diabetes Mellitus is a chronic disease with relatively clear diagnostic criteria and it has important implications for the quality of life of individuals that have the disease^13^, which may explain the high specificity of this self-reported information.

In this study, the prevalence of DM among residents of the urban area of the city of São Paulo was 8.0% (95%CI 6.1–10.6), whereas the prevalence of self-reported DM was 5.4% (95%CI 4.0–7.2), indicating that, despite the ease of collection and use of self-reported data, the prevalence of this chronic disease is underestimated when self-reported information is used. The same occurs when we compare the estimated prevalence of DM found in this study and the data from the VIGITEL-2008 of São Paulo, whose information is self-reported: 8.0% (95%CI 6.1–10.6) *versus* 6.5% (95%CI 5.3–7.7), respectively^16^.

An important limitation of validation studies on self-reported DM is the classification bias^11^. Only one measure of fasting glucose was used to classify the participants as individuals with DM in this study, while the ideal would be to repeat the test or to introduce the amount of glycated hemoglobin. Formerly only used for monitoring the glycemic control, glycated hemoglobin has the advantage of not being influenced by blood glucose fluctuations from one day to the next, and it is currently also indicated to confirm the diagnosis of DM or prediabetes^1^. However, because it is a population-based study, we could not measure fasting glucose again or another marker. Furthermore, the use of fasting glucose does not allow diagnosing all individuals with DM, as observed by Goto et al.^9^ However, the inclusion of the biochemical measure in studies increases the validity of the data and reduces the classification bias^13^. It is possible that subjects that were not diagnosed with DM but who self-reported the condition controlled their disease with their lifestyle, such as diet and physical activity, which are also regarded as supporting factors for glycemic control^1^.

Another possible source of error was the classification of all participants with fasting glucose ≥ 7.0 mmol/L (126 mg/dL) or use of oral medication or insulin as individuals with type 2 DM, with no stratification among the other types of DM or other cases that demand the use of oral hypoglycemic agents. This fact does not invalidate the results, since type 2 DM represents 90% to 95% of all DM cases^3^. Of the individuals with DM, 1.9% reported age of diagnosis under 18 years, an age group in which three quarters of the cases of type 1 or immune-mediated DM occur, though both did not use insulin, so they could not be classified as individuals with type 1 DM, and it may indicate the early onset of type 2 DM, which is increasingly being diagnosed at younger ages^1^.

Another limitation was the absolute number of individuals with DM (n = 82), which restricted the estimation of factors associated with the sensitivity of self-reported DM, producing large confidence intervals for some estimates and preventing us from using the multiple Poisson regression model. However, few variables were associated with the sensitivity of self-reported DM in the univariate analysis, which suggests that the impossibility of carrying out a multiple analysis is not a significant restriction. The same limitation was observed in a previous study conducted in the Netherlands: the Utrecht Health Project^13^.

The results obtained in this study provide evidence on the use of the self-reported DM information, often used in national and international studies^11,14,19^, validate the data in a representative sample of the city of São Paulo, and provide important prevalence estimates of DM for this city.

In conclusion, self-reported DM is valid, especially in older adults of the city of São Paulo. It is necessary to consider the age group of the study population in the analysis of this data, which may underestimate the prevalence of diabetes in the study population and reduce the power of association with other variables because of classification error. Therefore, the use of self-reported information will depend on the objectives of the study. In addition, the results show the need for the screening of DM in asymptomatic individuals that have one or more risk factors for DM, mainly in the adult population.

## Footnotes

a Ministério da Saúde (BR). VIGITEL Brasil 2013: vigilância de fatores de risco e proteção para doenças crônicas por inquérito telefônico. Brasília (DF); 2014.

b Alves MCGP, Escuder MML. Plano de amostragem do ISA - Capital 2008. [cited 26 May 2014]. Available from: http://www.fsp.usp.br/isa-sp/pdf/planoamostral2008.pdf

c Guenther PM, DeMaio TJ, Ingwersen LA, Berline M. The multiple-pass approach for the 24-hour recall in the Continuing Survey of Food Intakes by Individuals (CSFII) 1994-1996. Presented at the International Conference on Dietary Assessment Methods, January 1995; Boston, Mass.

d Sociedade Brasileira de Diabetes. Diretrizes da Sociedade Brasileira de Diabetes 2008. São Paulo; 2008 [cited 2016 Sept 16]. Available from: http://www.nutritotal.com.br/diretrizes/files/166--DiretrizesDiabetes2008.pdf

e Ministério da Saúde (BR). Rastreamento. Brasília (DF); 2010. (Cadernos de Atenção Primária, 29).
